# Kidney function in cachexia and sarcopenia: Facts and numbers

**DOI:** 10.1002/jcsm.13260

**Published:** 2023-05-24

**Authors:** Masatsugu Okamura, Masaaki Konishi, Javed Butler, Kamyar Kalantar‐Zadeh, Stephan von Haehling, Stefan D. Anker

**Affiliations:** ^1^ Berlin Institute of Health Center for Regenerative Therapies (BCRT) Charité – Universitätsmedizin Berlin Berlin Germany; ^2^ Department of Rehabilitation Medicine, School of Medicine Yokohama City University Yokohama Japan; ^3^ Department of Cardiology, School of Medicine Yokohama City University Yokohama Japan; ^4^ Baylor Scott and White Research Institute Dallas Texas USA; ^5^ University of Mississippi Medical Center Jackson Mississippi USA; ^6^ Department of Medicine, Division of Nephrology, Hypertension and Kidney Transplantation University of California Irvine School of Medicine Orange California USA; ^7^ Department of Cardiology and Pneumology University Medical Center Göttingen Göttingen Germany; ^8^ German Center for Cardiovascular Research (DZHK), partner site Göttingen Göttingen Germany; ^9^ Department of Cardiology (CVK) Charité – Universitätsmedizin Berlin Berlin Germany; ^10^ German Center for Cardiovascular Research (DZHK), partner site Berlin Berlin Germany; ^11^ Institute of Heart Diseases Wroclaw Medical University Wroclaw Poland

**Keywords:** Cachexia, Chronic kidney disease, Cystatin C, Protein‐energy wasting, Sarcopenia

## Abstract

Cachexia, in the form of unintentional weight loss >5% in 12 months or less, and secondary sarcopenia in the form of muscle wasting are serious conditions that affect clinical outcomes. A chronic disease state such as chronic kidney disease (CKD) often contributes to these wasting disorders. The purpose of this review is to summarize the prevalence of cachexia and sarcopenia, their relationship with kidney function, and indicators for evaluating kidney function in patients with CKD. It is estimated that approximately half of all persons with CKD will develop cachexia with an estimated annual mortality rate of 20%, but few studies have been conducted on cachexia in CKD. Hence, the true prevalence of cachexia in CKD and its effects on kidney function and patient outcomes remain unclear. Some studies have highlighted the concept of protein‐energy wasting (PEW) which usually include sarcopenia and cachexia. Several studies have examined kidney function and CKD progression in patients with sarcopenia. Most studies use serum creatinine levels to estimate kidney function. However, creatinine may be influenced by muscle mass, and creatinine‐based glomerular filtration rate may overestimate kidney function in patients with reduced muscle mass or muscle wasting. Cystatin C, which is least affected by muscle mass, has been used in some studies, and creatinine‐to‐cystatin‐C ratio has emerged as an important prognostic marker. A previous study incorporating 428 320 participants reported that participants with CKD and sarcopenia had a 33% higher hazard of mortality compared with those without (7% to 66%, *P* = 0.011), and that those with sarcopenia were twice as likely to develop end‐stage kidney disease (hazard ratio: 1.98; 1.45 to 2.70, *P* < 0.001). Future studies on cachexia and sarcopenia in patients with CKD are needed to report rigorously defined cachexia concerning kidney function. Moreover, in studies on sarcopenia with CKD, it is desirable to accumulate studies using cystatin C to accurately estimate kidney function.

## Definition of cachexia and sarcopenia

Cachexia and sarcopenia are serious conditions that affect the long‐term prognosis of patients, with weight loss and loss of muscle mass/strength as the main symptoms, respectively. Cachexia was defined at a consensus conference in Washington, USA, in 2006 as ‘a complex metabolic syndrome associated with underlying illness and characterized by loss of muscle with or without loss of fat mass’.^1^ Currently, the widely accepted diagnostic criteria are weight loss of at least 5% in 12 months or less, plus three or more of the following five criteria: decreased muscle strength, fatigue, anorexia, low fat‐free mass index, and abnormal biochemistry.^1^ On the other hand, sarcopenia is defined as a loss of muscle mass and strength, which can be divided into ‘primary sarcopenia’ due to aging and ‘secondary sarcopenia’ due to one or more obvious causes such as reduced activity, chronic disease, or poor nutritional status.^2^ The diagnostic criteria for sarcopenia include screening by questionnaire (SARC‐F) and clinical suspicion, followed by the assessment of muscle strength (grip strength and chair stand test), muscle quantity and quality (dual‐energy X‐ray absorptiometry, bioelectrical impedance analysis, computed tomography, and/or magnetic resonance imaging), and physical performance (gait speed, short physical performance battery, timed‐up and go test, and 400 m walk) to determine whether and how severe sarcopenia is.^2^

## Kidney function in cachexia and protein‐energy wasting

There is surprisingly little research on the prevalence of cachexia in patients with chronic kidney disease (CKD) and its impact on kidney function (Table 1). The presence of chronic diseases is considered a cause of cachexia and sarcopenia, and these include chronic wasting diseases such as cancer, heart failure, CKD, chronic obstructive pulmonary disease, autoimmune diseases, and chronic infectious diseases/sepsis.^1^, ^2^ von Haehling et al. estimate that the prevalence of CKD in the population is 0.1% and report that 50% of them have cachexia.^3^ They then estimate the number of patients with cachexia in CKD to be 190 000 in Europe, 80 000 in the United States, and 30 000 in Japan, and report a mortality rate of 20% in 1 year. McKeaveny et al. studied 106 adult patients on haemodialysis and reported that cachexia, diagnosed by the definition of Evans et al.,^1^ was present in 17 (16%) of them.^4^

**Table 1:** Characteristics of the studies (cachexia and protein‐energy wasting)

Author ‐ year	Country ‐ subjects	*N*, Age (mean ± SD), Gender (%), BMI (mean ± SD)	Kidney function	Description
de Mutsert et al. ‐ 2009^7^	The Netherlands ‐ End‐stage renal disease patients	1601, 59 ± 15, Men: 978 (61.1), Women: 623 (38.9), BMI 24.7 ± 4.1	Severe PEW (81) versus Moderate PEW (367) versus Normal nutrition (1153); rGFR 2.3 ± 2.2 versus 3.2 ± 2.9 versus 4.1 ± 3.0, *P* < 0.001	Severe PEW at baseline, as assessed with the 7‐point SGA, was independently associated with a 2‐fold increased mortality risk in 7 years of follow‐up. In time‐dependent analyses, this association was even stronger, 5‐fold, indicating that PEW was associated with a remarkably high risk of short‐term mortality.
von Haehling et al. ‐ 2016^3^	Europe, USA, Japan ‐ Estimates based on data	No detailed data	Estimates for the prevalence of cachexia in CKD; Prevalence in population 0.1%, Patients at risk 50%, Prevalence in patients at risk 50%, Patients in Europe 190 000, the USA 80 000, Japan 30 000, 1‐year mortality 20%	Given the high prevalence and very high mortality associated with cachexia, advances are urgently needed for patients worldwide.
Dai et al. ‐ 2017^8^	Sweden ‐ CKD patients	1031	Well‐nourished (711) versus Malnourished (according to SGA, PEW_SGA_, 320); Creatinine‐based eGFR 6.1 (0–68.8) versus 5.6 (0–11.4), *P* < 0.001, Cr (μmol/L) 664 (95–1017) versus 627 (403–917), *P* = 0.79	SGA, a valid assessor of nutritional status, is an independent predictor of all‐cause mortality both in CKD non‐dialysis and dialysis patients that outperforms non‐composite nutritional markers as prognosticator.
Hyun et al. ‐ 2017^9^	Korea ‐ Predialysis CKD patients	1834, 53.9 ± 12.2, Men: 1108 (60.4), Women: 726 (39.6)	Creatinine‐based eGFR Stage 1 (≥90) (231) versus Stage 2 (60–89) (339) versus Stage 3a (45–59) (327) versus Stage 3b (30–44) (405) versus Stage 4 (15–29) (418) versus Stage 5 (<15) (114); PEW 2.2 versus 4.4 versus 8.3 versus 6.2 versus 15.6 versus 24.6, *P* < 0.001	PEW increases with advanced CKD stage. PEW is independently associated with renal function, low total CO_2_, low physical activity, comorbid diabetes, and increased hs‐CRP in adults with predialysis CKD.
Pérez‐Torres et al. ‐ 2017^10^	Spain ‐ Patients attending the advanced CKD outpatient clinic	186, Men: 101 (54.3), Women: 85 (45.7) BMI 27.6 ± 5.1	Total versus Men versus Women; Cr 3.7 ± 1.1 versus 3.8 ± 1.3 versus 3.6 ± 0.9, Not significant, Cr clearance 17 ± 4 versus 18 ± 4 versus 17 ± 4, Not significant PEW (%) 56 (30) versus 23 (23) versus 33 (39), *P* < 0.001	Malnutrition was identified in Spanish advanced CKD patients measured by different tools. We consider it appropriate to adapt new diagnostic elements to PEW criteria.
Koppe et al. ‐ 2019^6^	Searched the publication in MEDLINE from February 2008 to September 2018	No detailed data	PEW prevalence increases when renal function declines, that is, from <2% in CKD stages 1–2 to 11–54% in CKD stages 3–5	The recent understanding of cachexia physiopathology during CKD progression suggests that PEW and cachexia are closely related and that PEW corresponds the initial state of a continuous process that leads to cachexia, implicating the same metabolic pathways as in other chronic diseases.
McKeavency et al. ‐ 2021^4^	United Kingdom ‐ Adult haemodialysis patients	106, 67.62 ± 13.18, Men: 76 (71.7), Women: 30 (28.3) BMI, median (IQR) 28.0 (23.0–31.3)	Cachectic versus Not cachectic; URR, median (IQR) 0.75 (0.72–0.81) versus 0.73 (0.68–0.77), *P* < 0.001, eGFR, median (IQR) 6.8 (5.5–6.8) versus 8.6 (6.85–10.7), Not significant	This is the first study to apply the defined characteristics of cachexia to a representative sample of patients receiving HD. Further, more extensive studies are required to establish a phenotype of cachexia in advanced CKD.

BMI, body mass index; CKD, chronic kidney disease; Cr, creatinine; eGFR, estimated glomerular filtration rate; HD, haemodialysis; hs‐CRP, high sensitivity C‐reactive protein; IQR, interquartile range; PEW, protein‐energy wasting; rGFR, residual glomerular filtration rate corrected for body surface area; SD, standard deviation; SGA, subjective global assessment; URR, urea reduction ratio.

On the other hand, there are some reports of CKD and protein‐energy wasting (PEW), a condition similar to cachexia. PEW is a concept proposed by the International Society of Renal Nutrition and Metabolism and the International Society of Nephrology in 2008 and is defined as the state of decreased body stores of protein and energy fuels (i.e., body protein and fat masses).^5^ The diagnostic criteria for PEW are three or more of the following four criteria: low serum chemistry (albumin, transthyretin, or cholesterol), low body mass, decreased muscle mass, and low protein or energy intakes, which are similar in many, if not identical to cachexia.^5^ Koppe et al. have therefore proposed positioning cachexia as a severe form of PEW.^6^ In a previous study of PEW and CKD, de Mutsert et al. divided patients with end‐stage renal disease into normal nutrition, moderate PEW, and severe PEW and reported that residual glomerular filtration rate calculated by the creatinine and urea clearance from a 24‐h urine sample was lower in the severe PEW group.^7^ Dai et al. reported a lower creatinine‐based estimated glomerular filtration rate (eGFR) in the PEW group than in the well‐nourished group, but there was no difference in creatinine.^8^ The PEW group includes more haemodialysis patients, and estimating the glomerular filtration rate of those patients as zero may have an impact on eGFR. Hyun et al. studied patients with CKD before the introduction of dialysis, and divided them into five groups according to creatinine‐based eGFR severity.^9^ The results showed that 9% of all 1834 subjects had PEW, with 2.2% in Stage 1 CKD, 4.4% in Stage 2, 8.3% in Stage 3a, 6.2% in Stage 3b, 15.6% in Stage 4 and 24.6% in Stage 5, by group, and that the prevalence of PEW tended to increase as the severity of CKD progressed. In a study of 186 Spanish patients with advanced kidney disease, Péres‐Torres et al. reported that PEW was present in 30% of all patients, 23% of men, and 39% of women, with a higher prevalence in women.^10^ In addition, one previous study incorporating 1031 CKD patients had shown that PEW was associated with increased mortality independently of other factors (risk ratio: 1.17, 95% confidence interval: 1.11 to 1.23, *P* < 0.0001).^8^

Patients with CKD often suffer from cachexia and PEW as a result of disease‐related factors. The aetiology of PEW encompasses anorexia, reduced energy and protein consumption, hypermetabolism, uraemia, metabolic acidosis, decreased physical activity, decreased anabolism, comorbidities (diabetes, chronic heart failure, coronary artery disease, peripheral artery disease, and depression), and dialysis.^4^, ^11^ Therefore, patients with end‐stage renal disease may have low kidney function as well as cachexia, a serious condition of PEW.

CKD is considered to be an important cause of cachexia, but few studies have reported on cachexia and CKD, and most studies have been on PEW and CKD. In patients with CKD, PEW prevalence increases as the severity of CKD progresses, and can have a negative impact on kidney function. More researches are needed in the future on the prevalence of strictly defined cachexia and its impact on kidney function in patients with CKD.

## Kidney function in sarcopenia

More studies on sarcopenia and kidney function in patients with CKD have been reported than on cachexia and PEW (Table 2). Isoyama et al. divided 330 haemodialysis patients into two groups according to muscle mass or strength, respectively, and reported that creatinine was lower in the group with reduced muscle mass and strength, although there were no significant differences in residual glomerular filtration rate calculated by the renal urea and creatinine clearances from a 24‐h urine collection.^12^ Yang et al. showed that serum creatinine and creatinine‐based eGFR were lower in the presence of sarcopenia in both diabetic and non‐diabetic patients.^13^ Hyun et al. conducted a large survey of 10 734 people in South Korea and divided them into normal, sarcopenia alone, obesity alone, and sarcopenic obesity groups.^14^ The results showed that creatinine‐based eGFR was lower in the order of these four groups and that the prevalence of CKD was also higher. Fukuda et al. studied sarcopenia and the ratio of the android fat mass divided by the gynoid fat mass (A/G), dividing them into four groups; non‐sarcopenic low A/G, sarcopenic low A/G, non‐sarcopenic high A/G, and sarcopenic obesity.^15^ There were discrepancies in creatinine‐based eGFR among the four groups, and the co‐occurrence of sarcopenia and a high A/G, referred to as sarcopenic obesity, resulted in the largest decline in creatinine‐based eGFR over 1 year. Moreno‐Gonzalez et al. reported that the prevalence of sarcopenia increases with more severe CKD when severity is classified using the formula, which estimates creatinine‐based eGFR, of the Berlin initiative study or the chronic kidney disease epidemiological collaboration.^16^ Soraci et al. reported that in elderly inpatients, those who died had a lower creatinine‐based eGFR and a higher prevalence of sarcopenia compared with those who survived.^17^ Although these studies use eGFR based on serum creatinine, Groothof et al. have suggested that creatinine‐based eGFR may be overestimated due to the muscle mass of the subjects.^18^ In settings of reduced muscle mass or muscle wasting, eGFR calculated by cystatin C may more accurately capture kidney function.^18^ Rizk et al. have reported that creatinine‐to‐cystatin‐C ratio is independently associated with mortality regardless of race or kidney function among 22 316 US veterans.^19^ Kusunoki et al. compared eGFR in community‐dwelling older people with and without sarcopenia.^20^ They reported that there were significant differences in cystatin C‐based eGFR with and without sarcopenia, but not in creatinine‐based eGFR. Similarly, Wilkinson et al. reported higher levels of cystatin C in sarcopenic patients with CKD than in non‐sarcopenic patients.^21^ Yoshimura et al. also reported that the use of creatinine‐based eGFR in stroke patients showed an opposite reduction in the prevalence of sarcopenia as kidney function declined.^22^ In addition, three previous studies showed that sarcopenia, particularly loss of muscle strength, was associated with increased mortality in patients with CKD.^12^, ^17^, ^21^ One previous study incorporating 428 320 participants reported that participants with CKD and sarcopenia had a 33% higher hazard of mortality compared with those without (7% to 66%, *P* = 0.011), and that those with sarcopenia were twice as likely to develop end‐stage kidney disease (hazard ratio: 1.98; 1.45 to 2.70, *P* < 0.001).

**Table 2:** Characteristics of the studies (sarcopenia)

Author, year	Country ‐ subjects	*N*, Age (mean ± SD), Gender (%), BMI (mean ± SD)	Kidney function	Description
Isoyama et al. ‐ 2014^12^	Sweden ‐ Incident dialysis patients	330, 53 ± 13, Men: 203 (61.5), Women: 127 (38.5)	Muscle mass; Appropriate versus Low; PEW 19 versus 43, *P* < 0.001, Serum creatinine 8.1 (5.3–11.7) versus 6.9 (4.2–10.4), *P* < 0.001 GFR calculated by renal urea and creatinine clearances 7 (4–9) versus 6 (4–10), *P* = 0.80 Muscle strength; Appropriate versus Low; PEW 16 versus 52, *P* < 0.001, Serum creatinine 8.3 (5.6–11.7) versus 6.46 (4.17–10.0), *P* < 0.001, GFR calculated by renal urea and creatinine clearances 7 (5–9) versus 6 (4–9), *P* = 0.27	Low muscle strength was more strongly associated with aging, protein‐energy wasting, physical inactivity, inflammation, and mortality than low muscle mass.
Hyun et al. ‐ 2016^14^	Korea ‐ General population	10 734, 49.5 ± 16.1, Men: 4691(43.7), Women: 6043 (56.3), BMI 22.21 ± 1.72	Normal (6325) versus Sarcopenia alone (1535) versus Obesity alone (16.0) versus Sarcopenic obesity (1152); Creatinine‐based eGFR 97.0 ± 15.5 versus 98.7 ± 17.7 versus 94.7 ± 16.6 versus 94.1 ± 19.4, *P* < 0.001, CKD (%) 2.3 versus 2.9 versus 3.4 versus 6.5, *P* < 0.001	Sarcopenic obesity was associated with CKD and high eGFR. Sarcopenia alone was associated with high eGFR. BMI, which was used as an operational definition for classifying both obesity and underweight, has long been recognized as an important indicator of nutrition and chronic debilitating disease.
Yang et al. ‐ 2016^13^	China ‐ Type 2 diabetes patients	1555	Without diabetes; Non‐sarcopenia (420) versus Sarcopenia (342); Creatinine‐based eGFR (mL/min) 111.49 ± 18.47 versus 100.95 ± 17.61, *P* < 0.001, Cr (μmol/L) 64.98 ± 12.86 versus 71.35 ± 15.15, *P* < 0.001 Diabetes; Non‐sarcopenia (585) versus Sarcopenia (208), Creatinine‐based eGFR (mL/min) 122.75 ± 29.20 versus 107.42 ± 30.95, *P* < 0.001 Cr (μmol/L) 61.57 ± 15.41 versus 77.31 ± 62.17, *P* < 0.001	Sarcopenia is associated with declining renal function, which in turn leads to lower eGFR and higher UACR in the non‐diabetic population and type 2 diabetics.
Fukuda et al. ‐ 2020^15^	Japan ‐ Type 2 diabetes patients	745, 64.6 ± 11.8, Men: 399 (53.6), Women: 346 (46.4)	Non‐sarcopenic low A/G (205) versus Sarcopenic low A/G (168) versus Non‐sarcopenic high A/G (287) versus Sarcopenic obesity (85); Creatinine‐based eGFR 71.5 ± 20.6 versus 76.5 ± 29.3 versus 67.9 ± 22.1 versus 68.9 ± 24.6, *P* = 0.003, Annual decline rate in creatinine‐based eGFR, −1.3 ± 3.1 versus − 2.4 ± 4.0 versus − 1.9 ± 3.7 versus − 4.0 ± 4.8, *P* < 0.001	Sarcopenic obesity evaluated through a whole‐body DEXA scan is significantly associated with decline in renal function in Japanese people with type 2 diabetes, even after adjustment for established risk factors of decline in renal function including eGFR, ACR and systolic blood pressure.
Moreno‐Gonzalez et al. ‐ 2020^16^	Austria, Germany, Israel, Italy, the Netherlands, Poland, Spain ‐ Community‐dwelling older adults	1420, 79.5 (77.0–83.0), Men: 616 (43.4%), Women: 804 (56.6%), BMI 27.0 (24.4–30.0)	Sarcopenia was more prevalent in participants with more advanced stages of CKD according to BIS (9.6% in stages 1 and 2 and 13.9% in stages 3a, 3b and 4, *P* = 0.042), and also according to CKD‐EPI (9.8% versus. 14.2%, *P* = 0.042).	Participants within poorer eGFR categories, irrespective of the equation used for its calculation, have a higher prevalence of sarcopenia and are more often severely sarcopenic.
Kusunoki et al. ‐ 2021^20^	Japan ‐ Healthy community‐dwelling elderly individuals	949, 73.2 ± 5.9, Men: 302 (31.8), Women: 647 (68.2), BMI 22.7 ± 2.9	Men: Normal versus Sarcopenia, Cre 0.87 ± 0.17 versus 0.90 ± 0.19, *P* = 0.374, eGFRcre 68.4 ± 13.6 versus 65.0 ± 13.2, *P* = 0.230, eGFRcys 72.5 ± 15.5 versus 62.7 ± 16.9, *P* = 0.003, eGFRcys/eGFRcre 1.07 ± 0.17 versus 0.96 ± 0.14, *P* = 0.002 Women: Normal versus Sarcopenia, Cre 0.65 ± 0.13 versus 0.66 ± 0.14, *P* = 0.670, eGFRcre 69.5 ± 13.8 versus 67.6 ± 14.1, *P* = 0.339, eGFRcys 75.8 ± 15.1 versus 69.1 ± 17.1, *P* < 0.001, eGFRcys/eGFRcre 1.10 ± 0.17 versus 1.02 ± 0.14, *P* < 0.001	Low eGFRcys (CKDcys) was more frequent in participants with sarcopenia than in normal participants. In the multivariate logistic regression analysis adjusted for complications (hypertension, diabetes, dyslipidaemia, liver disease, and heart disease), CKDcys was clearly related to sarcopenia based on AWGS 2019 while CKDcre was not.
Soraci et al. ‐ 2021^17^	Italy ‐ Elderly patients admitted to hospitals	504, Survived; 338, 79.6 ± 6.59, Men: 155 (45.9%), Women: 183 (54.1%), BMI 27.0 ± 5.02 Died; 166, 82.8 ± 6.23, Men: 86 (51.8%), Women: 80 (48.2%), BMI 25.9 ± 5.14	Survived versus Died; Creatinine‐based eGFR 48.4 ± 15.3 versus 42.5 ± 17.2, *P* < 0.001 Sarcopenia 51 (15.1%) versus 57 (34.3%), *P* < 0.001	Our study demonstrates that eGFR, anaemia, sarcopenia, cognitive and physical impairment variably interact in predicting long‐term survival of older patients discharged from acute care hospital.
Wilkinson et al. ‐ 2021^21^	United Kingdom ‐ Individuals with chronic kidney disease	8767, 62.8 ± 6.8, Men: 4033 (46.0), Women: 4734 (54.0), BMI 29.3 ± 5.2	Sarcopenic versus Non‐sarcopenic; Cystatin C 1.5 ± 0.5 versus 1.3 ± 0.4, *P* < 0.001	We found a probable sarcopenia prevalence of 9.7% among participants with reduced kidney function defined as an eGFR < 60 mL/min/1.73 m^2^; this prevalence was approximately double that seen in those without CKD. The presence of sarcopenia increases the risk of mortality and end‐stage renal disease.
Yoshimura et al. ‐ 2021^22^	Japan ‐ Stroke patients	813, 73.5 ± 11.8, Men: 423 (52), Women: 390 (48)	Creatinine‐based eGFR ≥90 (117) versus 60–89 (363) versus 30–59 (302) versus 15–29 (28) versus <15 (3); Sarcopenia (%) 60 (51.3) versus 173 (47.7) versus 144 (47.7) versus 8 (28.6) versus 0 (0.0), *P* = 0.116	Elevated creatinine‐based eGFR is associated with sarcopenia, dysphagia, and adverse rehabilitation outcomes after stroke. Our findings highlight the limitations of assessing renal function using creatinine levels in patients with sarcopenia; therefore, future studies using cystatin C are needed to validate our findings.

A/G, android to gynoid fat ration; ACR, urinary albumin‐to‐creatinine ratio; BIS, Berlin initiative study; BMI, body mass index; CKD, chronic kidney disease; CKD‐EPI, chronic kidney disease epidemiological collaboration; Cr, creatinine; Cre, creatinine; cys, cystatin C; CysC, cystatin C; DEXA, dual‐energy X‐ray absorptiometry; eGFR, estimated glomerular filtration rate; EWGSOP, the European working group on sarcopenia in older people; FNIH, the Foundation of the National Institutes of Health; GFR, glomerular filtration rate; PEW, protein‐energy wasting; SD, standard deviation; UACR, urine albumin: creatinine ratio.

Future studies on sarcopenia in patients with CKD will need to use cystatin C to accurately estimate kidney function. Although there have been several studies on sarcopenia and kidney function, most studies used creatinine to estimate eGFR. Creatinine is known to be influenced by muscle mass and the use of creatinine‐based eGFR in sarcopenia patients with reduced muscle mass may overestimate kidney function. There are, in fact, some studies that show the opposite trend to what might be expected, such as a decline in kidney function accompanied by a decline in the prevalence of sarcopenia. In contrast, cystatin C is known to be less affected by muscle mass and more accurate in estimating GFR than creatinine.^23^ Therefore, more studies of sarcopenia in patients with CKD using cystatin C may be warranted to more accurately estimated kidney function.

## Conclusions

The presence of cachexia and sarcopenia can adversely affect patients' prognosis, so they should be evaluated for CKD and other chronic diseases. However, although there are some studies about PEW and CKD, surprisingly little research has been conducted on cachexia and kidney function in CKD, and new studies are warranted. The use of creatinine in sarcopenia patients with reduced muscle mass or muscle wasting may overestimate kidney function and further studies using cystatin C will be required to accurately assess kidney function in patients with CKD.

## Conflict of interest statement

Masatsugu Okamura and Masaaki Konishi have no conflict of interest to disclose. Javed Butler reports consulting fees from Abbott, Adrenomed, Amgen, Array, Astra‐Zeneca, Bayer, Boehringer Ingelheim, Bristol Myers Squibb, CVRx, G3 Pharmaceutical, Impulse Dynamics, Innolife, Janssen, LivaNova, Luitpold, Medtronic, Merck, Novartis, Novo‐Nordisk, Roche, and Vifor. Kamyar Kalantar‐Zadeh has received honoraria and/or support from Abbott, Ardelyx, Astra‐Zeneca, Cara, Daiichi, DaVita, Fresenius, GSK, Haymarket Media, Kabi, Novartis, Novo‐Nordisk, Pfizer, Sanofi, Shire, Travere, and Vifor. Stephan von Haehling has been a paid consultant for and/or received honoraria payments from Astra‐Zeneca, Bayer, Boehringer Ingelheim, BRAHMS, Chugai, Grünenthal, Helsinn, Hexal, Novartis, Pharmacosmos, Respicardia, Roche, Servier, Sorin, and Vifor. Stephan von Haehling reports research support from Amgen, Boehringer Ingelheim, IMI, and the German Center for Cardiovascular Research (DZHK). Stefan D. Anker has received grants from Abbott Vascular and Vifor International; and has received personal fees from Abbott Vascular, Actimed, Vifor, Bayer, Boehringer Ingelheim, Brahms, Novartis, Servier, Impulse Dynamics, Cardiac Dimensions, and Thermo Fisher Scientific; all outside the submitted work.
